# Using Trees as a Natural Weather Station for Wind Pattern Forecasting Applied to Forest Firefighting

**DOI:** 10.3390/s25237205

**Published:** 2025-11-26

**Authors:** Virgínia Vieira Aires, Cleonilson Protasio Souza, Orlando Rocha Baiocchi, Ane Polline Lacerda Protasio

**Affiliations:** 1 Graduate Program of Electrical Engineering, Federal University of Paraíba, Joao Pessoa 58051-900, PB, Brazil; virginia.aires@estudante.cear.ufpb.br; 2 Department of Electrical Engineering, Federal University of Paraíba, Joao Pessoa 58051-900, PB, Brazil; 3 School of Engineering & Technology, University of Washington Tacoma, Tacoma, WA 98402, USA; baiocchi@uw.edu; 4 Coordination of Public Health, Federal University of Pernambuco, Recife 50670-901, PE, Brazil; anepolline@hotmail.com

**Keywords:** wind forecasting, wavelet transform, machine learning, climate modeling, temperature sensors, trees

## Abstract

With the increasing frequency and intensity of extreme weather events, there is a growing need to develop innovative and accessible methods for environmental monitoring. This work presents a solution based on natural trees equipped with a low-cost embedded system. The innovative idea is that measuring only internal temperature of a tree around its trunk, it is possible to evaluate some weather parameters, such as wind speed and direction. To evaluate this relationship, a multiscale decomposition technique (Discrete Wavelet Transform) and machine learning models (Random Forest, Gradient Boosting, SVM, and Linear Regression) were applied. Among the models tested, Random Forest achieved the best results, demonstrating high accuracy with an error of 6.60% over a continuous monitoring period of 47 days. As an important outcome, the result shows that the proposed tree-based solution is viable for weather monitoring in hard-to-reach places, particularly in the context of forest fire prevention.

## 1. Introduction

In recent years, countries such as the United States, Canada, and Brazil have shown a significant increase in the occurrence of forest fires. Facing a reality where the combination of natural factors, intensified by the effects of climate change, and human actions, such as illegal burning, contribute to the increase in the intensity of these events, as well as to the creation of ideal conditions for fire propagation. It is from this scenario that the need arises for the development of new approaches to environmental monitoring.

In the study conducted by Littell et al. [1], the correlation between the increase in forest fire records in North America and the causes of extreme climatic changes (prolonged droughts), the replacement of natural vegetation for agricultural expansion, and significant alterations in the topography of the studied regions is presented. In the case of Brazil, the scenario is not different. Fire outbreaks are present in all its biomes [2], as observed in the Cerrado and the Amazon, leading the country to reach record rates of incidence in 2024.

To better understand fire behavior during an extreme event, it is necessary to evaluate which environmental parameters most influence it. Wind, for instance, stands out as one of the main causes of the unpredictable propagation of flames. This occurs because its speed and direction are determining factors for the advance of fire, how far a spark can travel, and where it can ignite a new fire source. Given the importance of wind as a propagation factor, predicting this variable in real time has become an increasingly urgent necessity in areas characterized by dense forests, rugged terrain, and limited access.

Estimating the fire spread rate is one of the major challenges involved in environmental behavior modeling. One of the most commonly used approaches is the ten percent wind speed rule. It proposes that the flame spread rate corresponds to approximately ten percent of the average wind speed in open space. This rule provides a simple and quick initial estimate, especially in emergency situations. However, it presents limitations when applied in locations with moist vegetation or irregular terrain [3].

A remarkable example in the history of an extreme climatic event was the so-called Firestorm that occurred in Chile in 2017, where the combination of record temperatures with a dry and fire-prone climate generated a sequence of severe wildfires, causing great environmental impact and health issues due to intense smoke [4]. The scenario and its conditions not only hindered firefighting and control efforts but also reinforced the importance of predictive tools as fundamental support for emergency response teams.

Given this necessity, recent studies have sought to explore innovative approaches for environmental monitoring. Research has shown that plants and trees can be used as biotic elements in the development of natural detection and monitoring interfaces. For example, as sources of renewable energy in the form of bioelectricity produced from aerated plant microorganism fuel cells, using ornamental plant species such as Chlorophytum comosum, Chasmanthe floribunda, and Papyrus diffusus [5].

In addition to the direct effects of fires, it has been observed that changes in vegetation use and composition also influence fire occurrence and intensity. The study conducted by Cavalett et al. [6] showed that the cultivation of Eucalyptus for bioenergy purposes can contribute to climate mitigation but may also cause local environmental impacts, such as increased eutrophication and acidification. These results reinforce the need for a better understanding of the relationship between vegetation, land use, and fire behavior, highlighting the importance of new environmental monitoring tools.

In this context, the present study proposes an innovative alternative to the use of conventional meteorological stations: the application of living trees as natural monitoring platforms. These trees are equipped with temperature sensors installed at different heights, cardinal orientations, and depths within the trunk of the selected tree, allowing observation of how internal thermal variations occur within the trunk. This leads to the central hypothesis of the study: how these thermal variations reflect external meteorological conditions, such as wind direction and speed.

Thus, we propose the concept of a Natural Weather Station (NWS), which employs trees as elements for environmental data collection. The study seeks to evaluate the potential of these internal temperature measurements by associating mathematical analysis techniques to complement real time meteorological monitoring. The proposal aims to achieve a low cost and low energy consumption solution to be used in regions where meteorological infrastructure is scarce, in addition to contributing to the advancement of sustainable environmental observation technologies.

## 2. Related Work

The development of technologies aimed at environmental monitoring has advanced steadily in recent years. Solutions involving remote sensing, artificial intelligence, and physically coupled modeling have significantly improved the accuracy and capacity for observing forest fires and their consequences. At present, there is a wide variety of systems in use, ranging from satellite imagery and unmanned aerial vehicles (UAVs) to biologically integrated sensors designed for surveillance, early detection, and fire spread prediction.

Among the most widely used approaches are multisensor satellite-based systems, which show high efficiency in the process of detecting and segmenting areas at risk of fire. The study by Ali and Kurnaz (2025) [7] demonstrated that deep learning models, such as Convolutional Neural Networks (CNNs) and U-NET, optimized through satellite data from Sentinel-1, Sentinel-2, and Landsat, achieved an accuracy of 82 percent in fire detection. It also showed that deep learning-based methods outperformed traditional approaches based on spectral pattern analysis.

Similarly, Zhang et al. (2021) [8] proposed a model for detecting active fires using Sentinel-2 satellite imagery by combining the DCPA module with the HRVNetV2 architecture. The DCPA is known as an attention mechanism that allows the model to focus on the most relevant regions of the image, while HRNetV2 is responsible for maintaining high-resolution representations throughout the analysis process, preserving spatial details and the edges of burned areas. This combination achieved excellent performance, with an intersection over union (IoU) greater than 70 percent, and generated high-resolution outputs for areas up to 12,000 square kilometers in an estimated time of less than six minutes.

At the same time, the use of unmanned aerial vehicles (UAVs) for environmental sensing has been expanding, allowing high spatial and temporal resolution for the monitoring of forested and agricultural areas. Vanegas et al. (2018) [9] proposed the integration of UAVs with equipment such as RGB, multispectral, and hyperspectral cameras for detecting pest-induced stress in vineyards, demonstrating the potential of these platforms for environmental applications.

In the case of forest fires, UAVs have been employed to monitor burned areas, validate satellite imagery, and analyze fire behavior in agricultural systems practicing rotational shifting cultivation (RSC). This traditional practice generates combustion patterns that differ from those observed in large-scale wildfires.

In the study presented by Arunrat et al. (2024) [10], UAV images were used to assess fire dynamics and post-burn changes in the chemical and microbial properties of soil in northern Thailand. Rapid alterations in soil characteristics were revealed, followed by seasonal recovery influenced by monsoon cycles. By incorporating thermal imagery of the area, it was also possible to identify burning patterns and fire perimeters with reasonable accuracy across heterogeneous landscapes.

The results confirmed the efficiency of using UAVs in the study of fire regimes, especially in regions where detection through orbital platforms is not feasible. However, their use remains limited by factors such as flight autonomy, the need for qualified operators, and dependence on favorable weather conditions, which restricts their application in dense forest environments.

Another relevant advancement concerns the automatic detection of fires. Valero et al. (2018) [11] proposed the use of unsupervised Canny edge detection to map the boundaries of active fires based on thermal infrared imagery and heat points. The system demonstrated good performance in delineating combustion fronts and produced results compatible with geographic information system (GIS) applications, eliminating the need for human calibration for real-time operation.

In the area of fire behavior prediction, the use of coupled atmospheric models has contributed to significant progress. The development presented by Kale et al. (2024) [12] introduced a model adapted for the Himalayan region, integrating atmospheric, topographic, and fuel vegetation data into the WRF SFIRE model. The platform enabled the simulation of fire propagation, providing real-time results and generating alerts for users.

From an ecological and biological perspective, there have been reports on the ability of plants to function as environmental sensing systems. Azri et al. (2018) [5] demonstrated the generation of bioelectricity from plant microbial fuel cells (PMFCs) using ornamental species, proving that plants are capable of responding to environmental stimuli through electrochemical signals.

Although active monitoring systems such as satellites and UAVs have demonstrated great efficiency, they still require specific infrastructure, preventive maintenance, and continuous energy supply, which limit their use in remote environments. Therefore, the present study proposes a simplified and environmentally integrated approach, focusing on how trees respond to environmental conditions that affect their internal thermal states. It investigates how trunk internal temperature can serve as an indirect indicator of wind conditions, offering a low-cost alternative for real-time meteorological monitoring.

## 3. Experimental Setup

If this hypothesis of predicting wind speed and direction from internal tree temperatures was to be tested, then an experimental system would need to be developed for obtaining robust and reliable data. This configuration is composed of two main devices: a tree with temperature sensor nodes (the NWS tree) and an automatic weather station juxtaposed to the vicinity of the tree. The automatic weather station provides a reference and its data are used to establish comparisons between the internal tree temperature conditions and external environment parameters, such as wind speed and direction. In conclusion, the internal trunk temperature at respective positions in NWS and the weather station is recorded under hot ambient air conditions.

This section describes the system components and settings for each of the systems, including sensor specifications, the test environment, data collection process, and selection criteria to provide an overall picture of the experimental conditions that support our findings in this paper.

### 3.1. The Proposed Natural Weather Station

**Figure 1:**
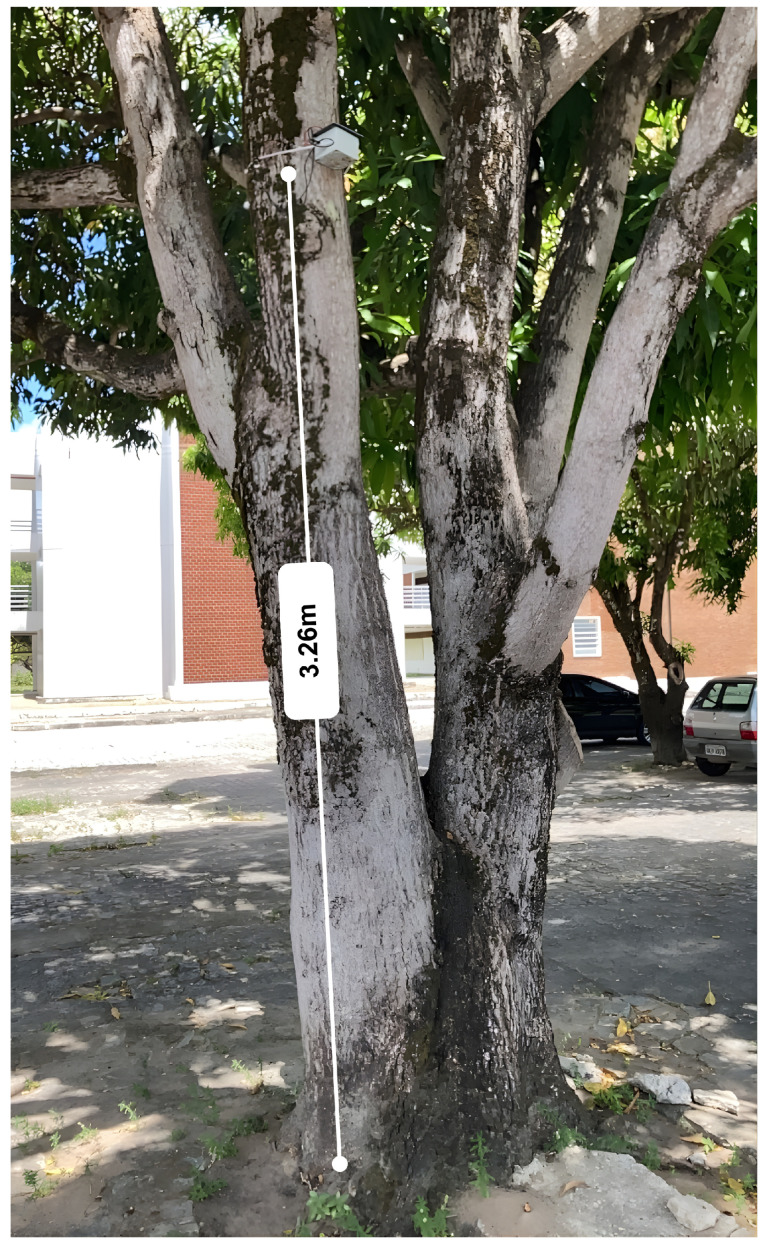
Original installation of the tree-based Natural Weather Station (NWS) on a mango tree, illustrating the system’s black enclosure and its position above the ground.

The designed tree-sensor-based NWS is a sensor-laden living tree with temperature measurement units, a microcontroller, and wireless radio. The sensors were buried straight into the bole at varying depths, heights, and cardinal positions (North, South, East, West) in order to acquire measurements and avoid external grounding effects as well as obtain internal thermal dynamic response with respect to external environmental influence such as solar radiation, shading, and wind. The tree-based NWS was tested on the campus of Federal University of Paraíba, Brazil (see Figure 1).

A healthy mango tree (*Mangifera indica*) was chosen for its stout canopy, girth, and commonality in the study area. This species was selected for the following reasons: its large trunks allowed the sensors to be repeatedly and safely inserted, without compromising the structural integrity of the tree; its dense foliage created different light and shade conditions during the day; and it has physiological stability under tropical conditions, which is suitable for testing whether internal thermal measurements are reliable.

It should be stressed here that the study was conducted with a single tree, which was in accordance with its exploratory and proof-of-concept character. Since the goal was not to derive statistically generalizable findings, rather to show that technically feasible inferences can be made from core trunk temperature, this was achieved. The specific setup will be broadened in future studies as it is replicated among species, environments, and time periods.

Internal trunk temperature was recorded with a DS18B20 digital temperature sensor (Maxim Integrated, San Jose, CA, USA) in the experiment. This sensor was selected because of its high accuracy (±0.5 °C from −10 °C to +85 °C), wide operating range (−55 °C to +125 °C), low power supply, and field-friendliness of installation. Operating via a digital protocol, the DS18B20 sends temperature data directly in digital form instead of as analogue signals, which reduces interference and eases data acquisition [13].

One of the advantages of the DS18B20 is that it uses the so-called 1-Wire protocol to communicate. This is a bus that allows several sensors to share a single data line, which makes wiring quite simple and easy to install multiple sensors in a complex environment. The sensors have unique digital addresses and can be identified separately even if several are patched in parallel [14].

A total of nine sensors were inserted through the tree trunk. They were equally spaced at 1 m, 2 m, and 3 m heights from the ground. Sensors were located at different heights and cardinal (N, S, E, W) directions and also inserted at three depths: 4.5 cm, 9.0 cm, and 13.5 cm, thus determining a radial-vertical grid of temperature measurement points. This setup allowed the study of thermal gradients caused by wind effect, sun exposure, trunk heat inertia, and bark insulating layers.

**Figure 2:**
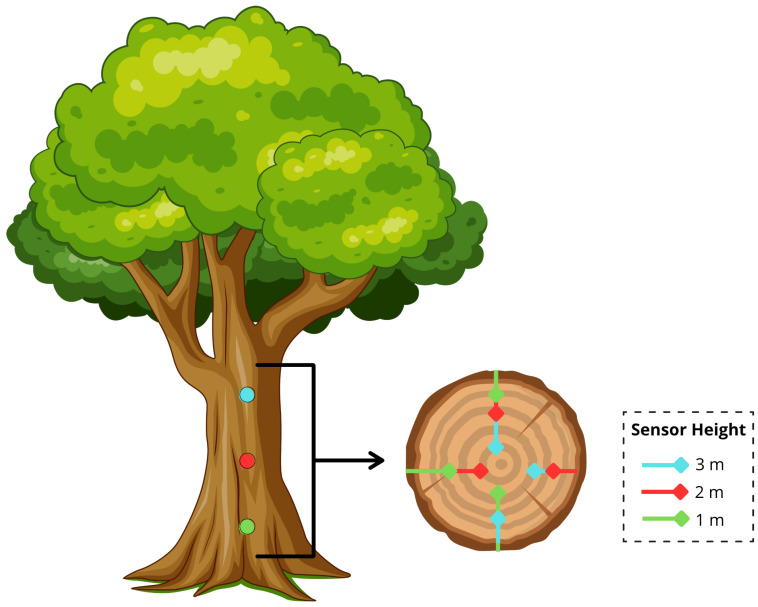
Spatial distribution of DS18B20 temperature sensors around the trunk at different heights, orientations, and insertion depths.

The exact location of each sensor is provided in Table 1 while their positions around the tree trunk are depicted in Figure 2. The multiple data depths were designed to estimate how fast and near the surface temperature deviations are forced to penetrate into the trunk, and whether this pattern responds to changes in wind intensity or direction.

**Figure 3:**
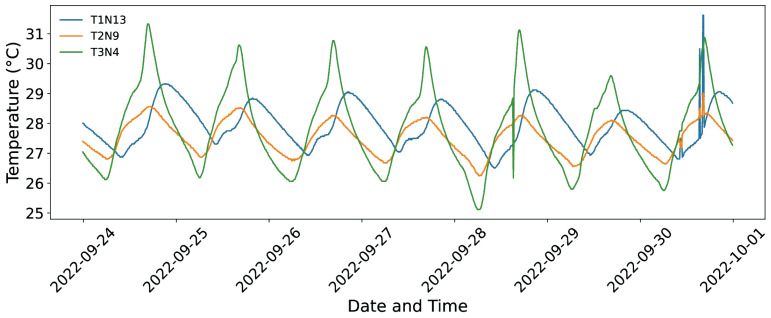
Example of one-week temperature time series (24 September–1 October 2022) from sensors installed at different positions on the tree trunk.

Temperatures were logged from the start to the finish of the experiment. Figure 3 is an example of a one-week period (24 September to 1 October 2022) chosen to show in more detail how the behavior evolves during the experiment. The diagram illustrates temperature differences between various depths, elevations, and orientations of the trunk. While the sensors are relatively parallel and have similar trends overall, it is evident that thermal responses are affected by depth of burial, exposure, and external weather conditions.

**Figure 4:**
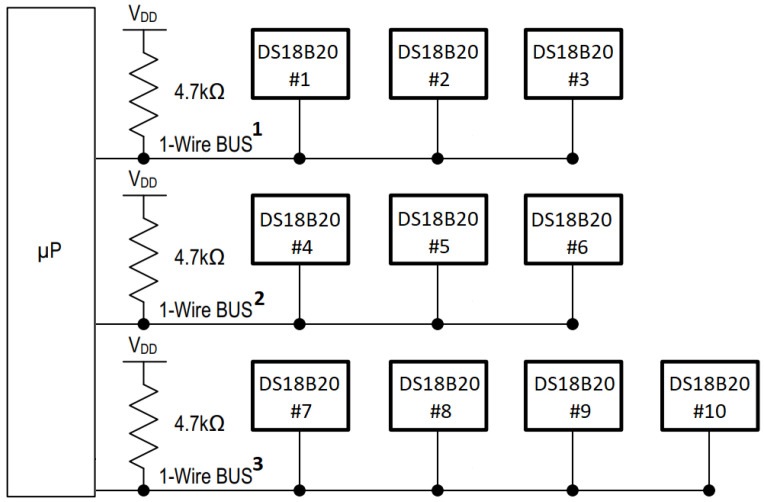
1-Wire buses interconnecting arrays of DS18B20 temperature sensors at each height and an external reference probe. The symbols “#1”–“#10” indicate the identification numbers of the sensors.

The DS18B20 temperature sensors were connected in arrays of three sensors, totaling three 1-Wire buses: 1-Wire Bus 1, 1-Wire Bus 2, and 1-Wire Bus 3, as shown in Figure 4. 1-Wire Bus 1 connected the set of sensors at 1 m height, 1-Wire Bus 2 the sensors at 2 m, and 1-Wire Bus 3 the sensors at 3 m. On 1-Wire Bus 3, an additional sensor measured the external temperature. Up to four sensors were attached per 1-Wire bus due to the drive capability limitation of the microcontroller I/O pin.

The system employs an ARM Cortex-M0-class microcontroller embedded on a commercially-available board (Adafruit Feather M0, Adafruit Industries, New York, NY, USA) paired with the 915 MHz LoRa radio module. With this configuration, it can read the sensors, process the values, and send packets including temperature readings. The embedded system is supplied by a solar panel, battery, and BMS, enabling energy autonomy in non-electrical powered zones. This supports the sustainability concept and is conducive to the continuous monitoring over the whole observation period, even in remote forest areas.

The data is sent to a cloud-based storage system on the LoRa network, enabling long-range wireless communication with low power. The packets transmitted by the system are received and processed in a LoRa gateway (The Things Network, v3.19, Amsterdam, The Netherlands), which works as a bridge to the Internet—it is placed 500 m from our system on the roof of a building. The data are transmitted to the storage system by the gateway and preprocessed for modeling weather variables.

**Figure 5:**
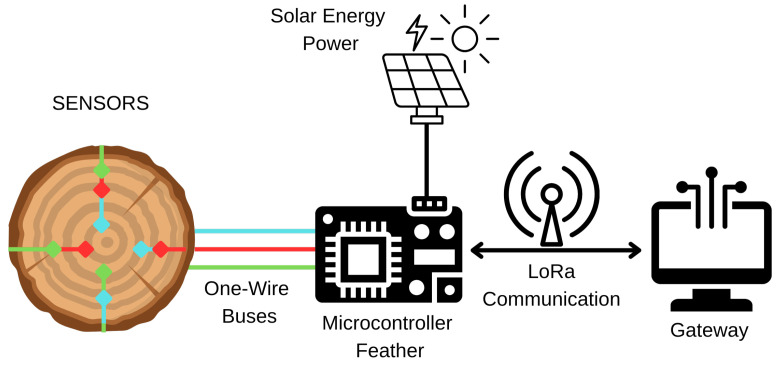
LoRa-based communication between the tree-sensor node and the gateway for cloud data transmission. The colors on the left correspond to those shown in Figure 2, representing sensor heights at 1 m, 2 m, and 3 m.

The overall system architecture for monitoring tree trunk temperatures and uploading them to the cloud is depicted in Figure 5. It provides round-the-clock, real-time, and high-resolution records of each interior temperature inside the tree.

### 3.2. Weather Station

The environmental data were collected from the automatic weather station of the Center for Alternative and Renewable Energies (CEAR) of the Federal University of Paraíba (UFPB), Brazil. The weather station is positioned about 290 m from the intended tree-based NWS, which should be close enough to reliably characterize the atmospheric conditions experienced by equivalent monitor trees.

**Figure 6:**
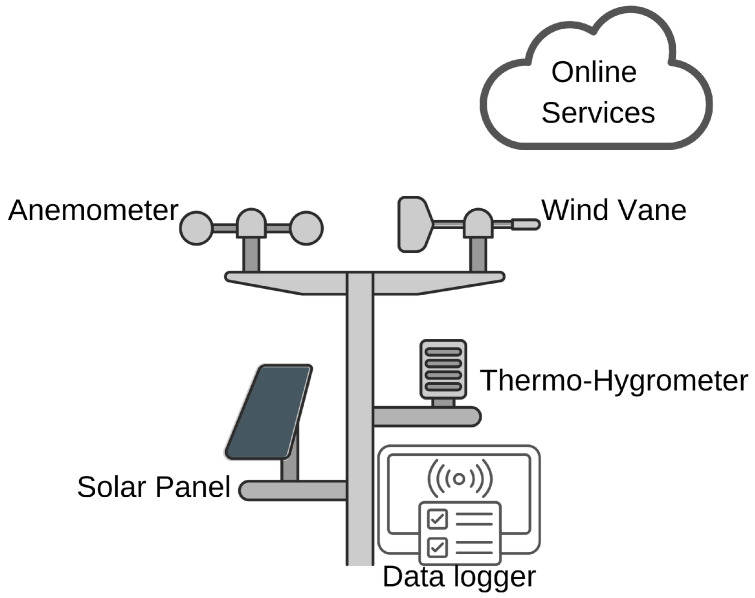
Automatic weather station (CEAR/UFPB) used as reference for wind speed and direction.

In this respect, two elements from the automatic weather station are particularly relevant to our work: the anemometer (to measure wind intensity) and the wind vane (for wind direction), as depicted in Figure 6. The raw data obtained from the AWS are saved on a data logger and an online storage site. These data have served as the reference variables to check the reliability of the predictions from models based on trees’ thermal time series.

**Figure 7:**
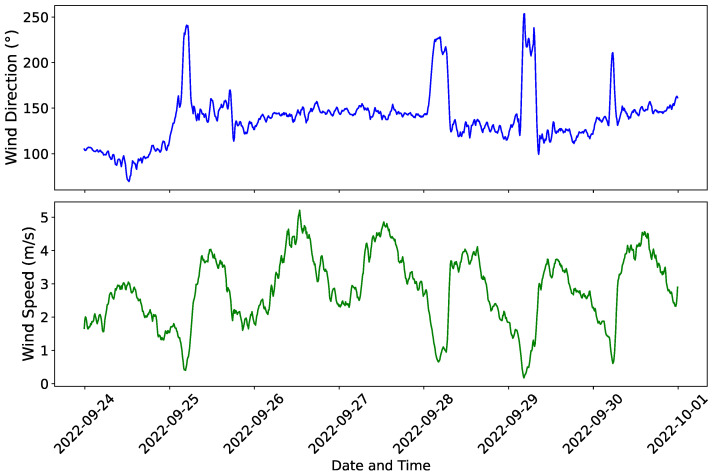
Example of one-week (24 September–1 October 2022) of wind direction (**top**) and wind speed (**bottom**) measured by CEAR/UFPB automatic weather station during a representative week.

The wind direction (upper graph) and the wind speed time series (lower graph), measured by CEAR/UFPB meteorological station, during a typical one-week period of days [from September 24 to 1 October 2022], are presented in Figure 7. The complete dataset is 47 days long; we picked this subset to represent the wind behavior during most of the campaign. Daily patterns of wind strength are evident, generally peaking in the warmest part of the day, and there is marked direction variation. These data are necessary to assess the effect of the wind on tree thermal dynamics.

In addition to wind, the station records other weather components such as air temperature and humidity through a thermo-hygrometer, which extends its possible applications for future works.

To prevent the rapid depletion of the battery and ensure complete charging, a data logger is incorporated to log all the measurements over time from which it automatically sends measurements online, transmitting them when required for visualizing remotely or integrating with other systems. This configuration supports having accurate and recent time series that are fundamental for the forecast of wind speeds and directions.

## 4. Data Processing and Models Used

As the tree-structured NWS models will only be properly exercised when trained on, a stage was developed as a first pass to pre-process data for that and perform this training. This step is for preprocessing, cleaning, and transformation of the input data for analysis. In this limited study, NWS tree-based measurements and measurements from a nearby station were temporally aligned to make valid comparisons. Although we use the in situ measurements as the core of this study, estimation can be considered a point of reference to Cal/Val (calibration/validation) products based on satellite remote sensing, making an effort for multi-source environmental monitoring.

Mathematical methods were subsequently employed to capture characteristics from the temperature time series using DWT, with which signals are decomposed into several temporal scales. This facilitates the detection of long-term trends as well as rapid short-term fluctuations, necessary to depict the wind-related features in data.

The data preprocessing steps along with the ML models for predicting wind speed and direction are discussed in subsequent sections. The aim is to demonstrate the process that raw temperature data underwent to be used as model input for the training and testing.

**Figure 8:**
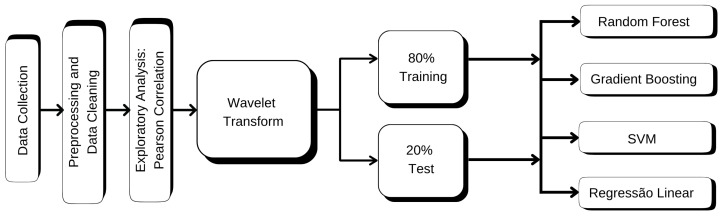
Overview of the proposed methodology: data acquisition, preprocessing, feature extraction via DWT, and machine learning modeling.

Figure 8 shows the summary of prototype design, including data preparation and model building.

### 4.1. Data Collection and Processing

The data in this study came from the tree-based NWS installed in a mango tree (Mangifera indica) at the Center for Alternative and Renewable Energies—CEAR/UFPB. Criteria for the selection of this tree included accessibility to the trunk, degree of branching and exposure of the canopy to wind, and distance from the automatic weather station. The study was conducted only on a single tree to run a pilot experiment and test the applicability of the proposed approach.

The tree-based NWS temperature sensors worked continuously for 47 days, gathering temperatures every 10 min and detecting thermal changes between day and night. Meteorological data (wind speed and direction) recorded by the automatic weather station installed in proximity to the monitored tree were also collected at the same time. Wind data were synchronized temporarily with the tree’s thermal measurements such that comparison analyses encompassed series in time.

The raw data were arranged into a database of tree temperature, its own timestamp, and the associated wind. The first step was to temporally standardize the records, such that timestamps were correctly ordered and there was no missing time between two consecutive items. In addition, a simplistic local averaging procedure was implemented to fill in any missing values: when an entry of the time series is missing, it is replaced by the average of itself and two nearest neighbors from that variable (this process leaves unchanged overall statistical properties). The tensor of temperature readings from the sensors was set as explanatory (input) variables, while wind speed and direction were regarded as target (forecasting) variables in the models.

Finally, in the preprocessing phase, all the temperature values were normalized (in other words, they were put on a scale that is related to an average temperature usually between 0 and 1 or with mean equal to 0 and standard deviation equal to one). This de-correlation restricts sensors with large absolute values from dominating the learning of the model.

For example, if one sensor measures a temperature between 28 °C and 32 °C, another between 20 °C and 22 °C, obviously the first sensor thus shows bigger numerical values. Without normalization, the model could be biased to be more sensitive to this sensor purely due to its scale and not because it actually contains more meaningful information. Normalization scales the readings of both sensors only to make them contribute in the same way during learning using a value between zero and one.

In addition to the original thermal variables, new derived variables called Temp Gradients were created to represent the gradient of temperature, i.e., the difference between temperatures measured at different nodes on the trunk. This feature is introduced in order to capture thermal variabilities related to external perturbations, such as variation of wind circulation around the tree and consequently adding more information for the predictive models.

### 4.2. Discrete Wavelet Transform

Environmental time series that are taken across time, such as temperature fluctuations, can be volatile and are not stable throughout the period or for any two different points in time. Thus, there is a need for techniques that can be used to analyze these changes across various time scales. One of these techniques is Discrete Wavelet Transform (DWT) [15].

In the present work, the temperature collected along the tree trunk was converted to wavelet-based domains, transforming it to generate representations that describe slower trends and faster variations inherent in the signal. For that, we used a wavelet from the Daubechies family of order 4 (db4) due to its effectiveness in the detection of small variations in data [16]. The decomposition of the temperature series is made by DWT into two kinds of information:

- Approximation coefficients (cA): overall behavior of temperature over time; it describes the average and trend of time series (Equation (1));
- Detail coefficients (cD): they emphasize more rapidly varying, short-duration features such as spikes and the sharp turning up of an edge (Equation (2)).

The mathematical model of DWT decimation for a discrete signal x[n] is denoted as

(1) $$cA_{j}\left[n\right]=\sum_{k}x\left[k\right]\cdot \phi_{j,k}\left[n\right]$$

(2) $$cD_{j}\left[n\right]=\sum_{k}x\left[k\right]\cdot \psi_{j,k}\left[n\right]$$

In those equations, $\phi_{j,k}$ and $\psi_{j,k}$ are the scaling (father) and wavelet (mother) functions at scale *j* and location *k*. The approximation coefficients, $cA_{j}\left[n\right]$, capture low-frequency components (those corresponding to slow variations) of the input sequence, while the detail coefficients captured in $cD_{j}\left[n\right]$ integrate high-frequency contents (fast changes).

**Figure 9:**
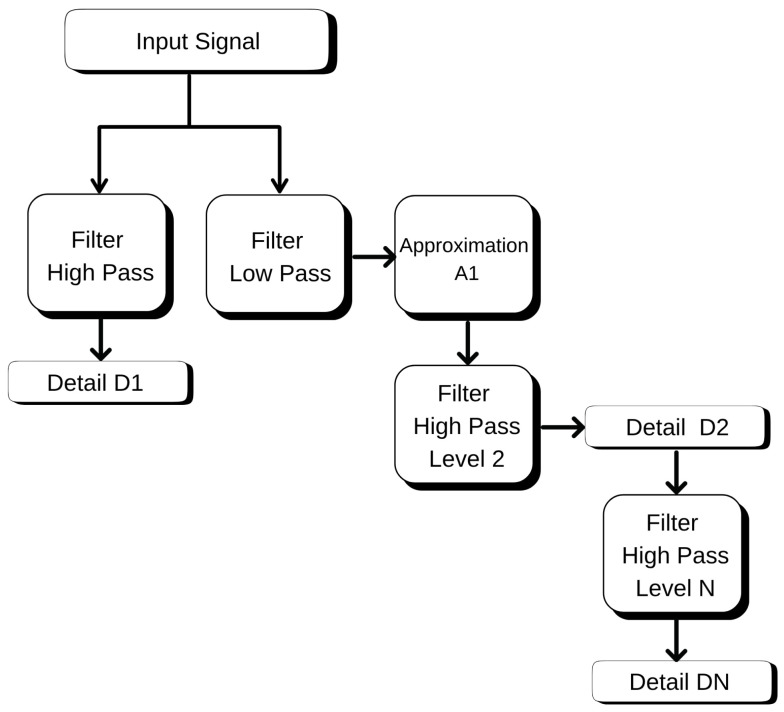
Discrete Wavelet Transform (DWT) decomposition of trunk temperature into approximation (cA) and detail (cD) coefficients.

At the conclusion of the decomposition, each time series was decomposed into four levels of detail, giving rise to one cA and four cD, as plotted in Figure 9. Effectively, that means observing the same signal in different resolutions as if with a magnifying glass of various zooming factors.

Following this transformation, the coefficients of downscaling and wind speed data as well as direction were subjected to correlation analysis. This aided the identification of which aspects of the temperature signal were most related to meteorological events. The coefficients that presented stronger correlation with wind behavior were used as predictive variables in the models proposed in this paper. Thus, the DWT contributed to transforming raw data into features that could be exploited by machine learning algorithms.

### 4.3. Models Developed

Four machine learning algorithms were trained to predict wind speed and direction based on the DWT-transformed features along with the original temperature variables: random forest (RF), gradient boosting, support vector machines, and linear regression. These models were selected for their capability to learn from noisy, non-linear environmental data and for providing a compromise between interpretability and performance.

The data were divided into 80% training and 20% testing. To prevent overfitting, when a model trains well but generalizes on test data poorly, the hyperparameters were tuned using the GridSearchCV framework with cross-validation. This strategy’s purpose was to avoid model over-fitting since all three classifiers tested are ensemble methods (RF and GB) or a non-linear classifier such as SVM with a radial basis function kernel [17].

Evaluation of each model was based on three performance criteria that are widely used in forecasting (and environmental modeling). These measures were chosen as they are commonly used and offer complementary views on model performance.

- Mean Squared Error (MSE): measures the average of the squares of the prediction errors. It is defined as Equation (3):
  (3) $$\mathrm{MSE}=\frac{1}{n}\sum_{i=1}^{n}{\left(y_{i}-{\hat{y}}_{i}\right)}^{2}$$
  where $y_{i}$ is the *i*-th observed value, ${\hat{y}}_{i}$ is the *i*-th predicted value, and *n* is the number of observations. By doing so, bigger deviations are more heavily penalized so that a lower MSE means better predictive performance.
- Coefficient of Determination ($R^{2}$): the amount of variance in the observed data that is accounted for by the model. It is given by Equation (4):
  (4) $$R^{2}=1-\frac{\sum_{i=1}^{n}{\left(y_{i}-{\hat{y}}_{i}\right)}^{2}}{\sum_{i=1}^{n}{\left(y_{i}-\bar{y}\right)}^{2}}$$
  where $\bar{y}$ is the average of observed values. $R^{2}$ values above 0 and below 1 are acceptable, and excellent performance in environmental studies has been related to $R^{2}$ greater than 0.70, even better when larger than 0.85.
- Mean Absolute Percentage Error (MAPE): describes the average prediction error as a percentage of the observed value. It is computed as Equation (5):
  (5) $$\mathrm{MAPE}=\frac{100}{n}\sum_{i=1}^{n}\left|\frac{y_{i}-{\hat{y}}_{i}}{y_{i}}\right|$$
  This measure is an intuitive scale-free definition of accuracy. MAPEs below 20% are typically considered good, with values less than 10% being excellent.

The forcing of all models was done with the same input mesh for wind speed and wind direction to make clear comparisons.

The models and their specifications are presented in the following sections:

#### 4.3.1. Random Forest (RF)

**Figure 10:**
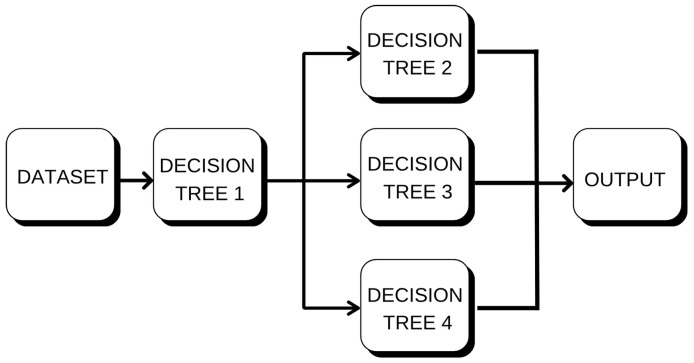
Random Forest model: ensemble of decision trees used to predict wind speed and direction.

Random Forest was the first model assessed, which is a method to create multiple decision trees using random samples of training data and predictors as shown in Figure 10. A prediction is given by each tree, and the predictions are averaged to obtain the final output, which is a common strategy in regression applications [18]. For each tree, the model produces its prediction, and the ensemble model output is an average of them as defined in Equation (6):

(6) $$\hat{y}=\frac{1}{N}\sum_{i=1}^{N}T_{i}\left(x\right)$$

where $T_{i}\left(x\right)$ is the prediction of the *i*-th tree and *N* is the total number of trees in the ensemble. This method, namely bootstrap aggregation or bagging, helps decrease the variance and hence results in robustness to overfitting.

Random Forest was shown to be an appropriate solution for the purposes of this investigation since it is able to model non-linear associations, noise resistance, and reporting variable importance. The model was first evaluated using the default parameters and fine-tuned by employing GridSearchCV with cross-validation. We tuned the key hyperparameters—number of estimators (trees), max depth of tree splits, and min samples to split.

#### 4.3.2. Gradient Boosting (GB)

**Figure 11:**
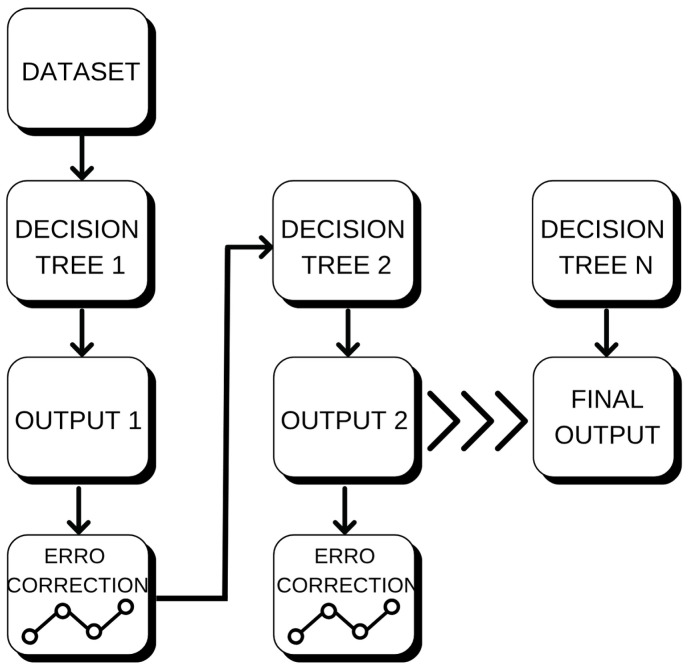
Gradient Boosting model illustrating sequential correction of residuals at each iteration.

GAn example of this kind of learning would be Gradient Boosting (GB), which is also using decision trees as its base classifier; however, GB learns the model in an iterative way unlike RF. The construction of every tree is done by looking at the remaining errors after iteration up to that point of the ensemble and in a way that reduces them, (this process takes place following the negative gradient of the loss function [19]), see Figure 11. At each iteration *m*, the model adapts the prediction function as in Equation (7):

(7) $$F_{m}\left(x\right)=F_{m-1}\left(x\right)+v\times h_{m}\left(x\right)$$

Here, $F_{m-1}\left(x\right)$ is the ensemble prediction up to now, $h_{m}\left(x\right)$ is a new weak learner (here we use a shallow regression tree), and ν is the learning rate determining how much each iteration contributes.

This iterative learning approach allows GB to achieve great predictability, but also it is more prone to hyperparameter tuning.

GB was particularly adept at capturing intricate non-linear temperature–wind patterns, but care had to be taken against overfitting.

#### 4.3.3. Support Vector Machines (SVM)

**Figure 12:**
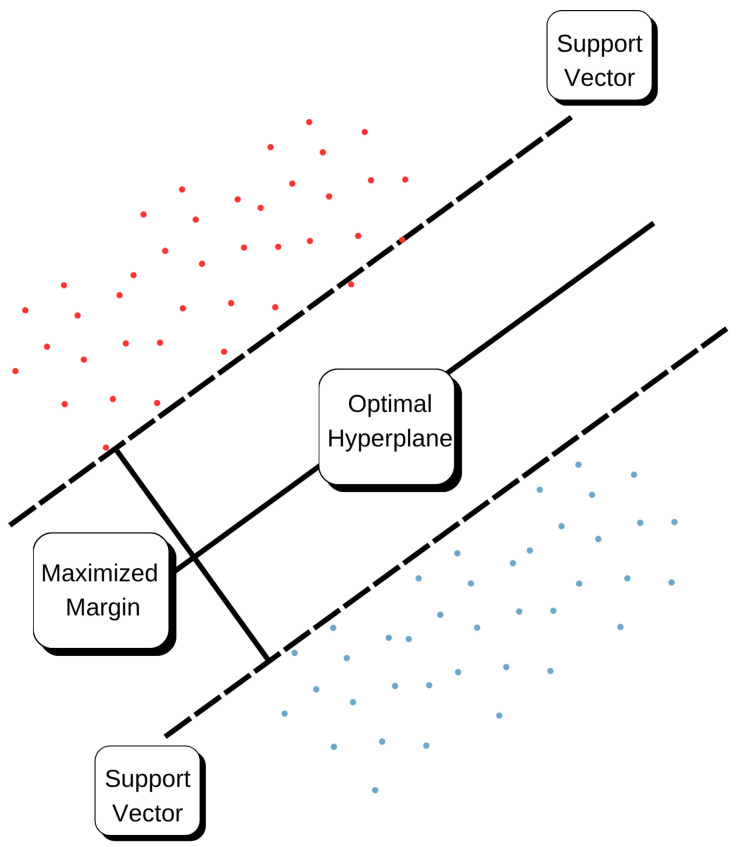
Support Vector Regression (SVR) model with ϵ-insensitive loss function and kernel mapping. The red and blue points represent data samples on opposite sides of the optimal hyperplane. The solid line indicates the optimal hyperplane, and the dashed lines denote the ϵ-margin boundaries. Points lying on the margins correspond to the support vectors.

For the regression problem, using the SVM model leads to the Support Vector Regression (SVR) [20] whose structure is depicted in Figure 12. The goal of SVR is to search for a function that fits the observed data in ϵ precision and at the same time makes the model as flat as possible. The fitting function is Equation (8):

(8) $$f\left(x\right)=w^{T}\phi \left(x\right)+b$$

Here, ϕ(·) is a kernel function that lifts input data into a higher dimensional feature space, *w* is the weight vector, and *b* is the bias term. The model complexity is determined by the regularization parameter, *C*, which tunes the trade-off between how flat (i.e., how large margin) our model should be and how much errors we are willing to tolerate.

In this study, two types of kernel function were evaluated:

- Linear Kernel that tends to explicit proportionality.
- Radial Basis Function (RBF) kernel, which is capable of learning nonlinear patterns in data.

Hyperparameters *C*, ϵ, and γ were tuned using cross-validation grid search. SVR is computationally intensive, but because we were interested in modeling nonlinear dynamics, this approach became feasible for environmental forecasting purposes.

#### 4.3.4. Linear Regression (LR)

For baseline comparisons, Linear Regression (LR) was used. It is based on the linear relationship between both of the input features, temperature and wavelet coefficients (of different scales), and output variables wind speed, wind direction [21]. The prediction of the model is given by Equation (9):

(9) $$\hat{y}=\beta_{0}+\sum_{i=1}^{p}\beta_{i}\times x_{i}$$

Here $\beta_{0}$ is the intercept and $\beta_{i}$ are the regression coefficients of each predictor $x_{i}$.

The observables and predictors of the models used for estimation are similar, and coefficients in them are estimated by minimizing the sum of squared residuals, which is the observed minus predicted value.

However, LR merely assumes linear responses and is easy to understand in the sense of parameter analysis, unlike the ARS model, which could well represent nonlinear interactions/abrupt transitions that usually happen in environmental systems. However, it is a necessary baseline to quantify the performance gained from more powerful models.

## 5. Results

After completing the data preprocessing and the development of the prediction models for training and testing, this section presents the main results obtained. Initially, the coefficients generated from the TWD are discussed, followed by the results of the models for wind speed and direction prediction.

This analysis highlights how the temperature variations recorded by the Natural Weather Station, based on trees, behave in relation to the actual measurements obtained from the meteorological station of the Federal University of Paraíba.

### 5.1. Discrete Wavelet Transform Coefficients

The time series after decomposition through the TWD resulted in two main sets. The first set corresponds to the approximation coefficients (cA), which show the long-term and generalized behavior of the signal, and the second set corresponds to the detail coefficients, responsible for capturing more localized and short-term variations.

**Figure 13:**
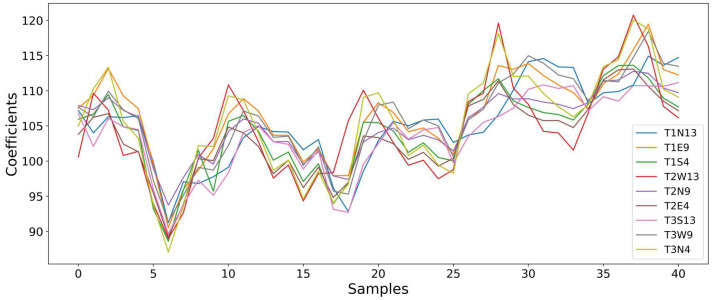
Comparison among approximation coefficients (cA) from multiple sensors showing coherent thermal dynamics within the trunk.

The similar interval among the sensors indicates a stable and approximately uniform thermal distribution across all measured points of the trunk. This correlation can be observed in Figure 13, where the behavior of the curves follows the same pattern throughout the analyzed samples.

On the other hand, when the detail coefficients are analyzed, a behavior with lower influence on the temperature data before processing can be observed. The first level coefficients ($cD_{1}$) exhibited high variability among the sensors, reflecting specific responses to local disturbances, such as direct sunlight incidence, greater shading, or other short-term environmental influences. It is verified that this variability tends to decrease in deeper decompositions, such as in $cD_{2}$ and $cD_{3}$, showing that the higher frequencies have little interference with the measured temperature values.

To confirm the potential of the TWD coefficients, both approximation and detail, in relation to the variables to be predicted, wind speed and direction, the Pearson correlation test was applied. The cA coefficients presented higher correlations with wind direction, ranging from 0.4 to 0.5, and lower correlations with wind speed, from 0.1 to 0.2, as shown in Table 2. On the other hand, the detail coefficients, at all decomposition levels, presented weak correlations, close to zero or negative, for both wind variables.

Based on the consistent and significant results, the approximation coefficient was chosen as the input for the predictive models. Although the detail coefficients were not used in this study, they remain relevant for complementary analyses and may be useful in future research.

### 5.2. Wind Speed Forecast

The wind speed prediction was developed according to the methodology already explained, in which the TWD coefficients with the highest Pearson correlation were used as input for the machine learning models. The approximation coefficients showed higher correlations; therefore, the results obtained for this variable presented smaller errors when compared to the second variable, direction.

Table 3 presents the evaluations obtained using the MSE, $R^{2}$, and MAPE metrics for the tested models: Random Forest (with and without optimization), Gradient Boosting, Support Vector Machine, and Linear Regression.

Among the tested models, the Random Forest with optimized hyperparameters presented the best result in terms of accuracy and stability, showing the lowest error values (MSE and MAPE) and the highest $R^{2}$ value, meeting all criteria commonly accepted in environmental modeling (MAPE < 20%, $R^{2}$ > 0.70). This advantage is related to the model’s ability to reduce overfitting and better identify the nonlinear connections between thermal variations and wind speed. Similar results were reported by Aminuddin et al. [22], who showed that RF and GB algorithms are highly robust for environmental time series prediction, with RF being more accurate for nonlinear or noisy data.

Similarly, Ekinci et al. [23] observed the stability of RF and GB model performance for medium and long term predictions. The possibility of hyperparameter optimization and data scaling also contributed to the results obtained in the model proposed in this study.

The GB model achieved competitive performance, with $R^{2}=0.803$ and MAPE = 20.96%, although it showed greater sensitivity to abrupt thermal variations and data noise. This behavior is consistent with the findings presented by Oyucu et al. [24], who observed that GB achieves high accuracy but is more susceptible to temporal variability. Nevertheless, it is the most advantageous method when computational and temporal efficiency are desired, proving to be faster than RF.

The results obtained from the SVM model can be considered intermediate, with $R^{2}=0.776$ and MAPE = 22.33%, but with a tendency to smooth the peaks in the data curves, reflecting its greater difficulty in representing nonlinear changes. Similar results were found in the studies by Cao et al. [25], indicating the need for fine tuning of the kernel and parameters to improve its performance in complex environmental signals.

The Linear Regression model presented the worst performance among all those analyzed, with low results of $R^{2}=0.544$ and MAPE = 38.27%, confirming the limitation of linear models in capturing more complex interactions defined between wind and temperature [24].

**Figure 14:**
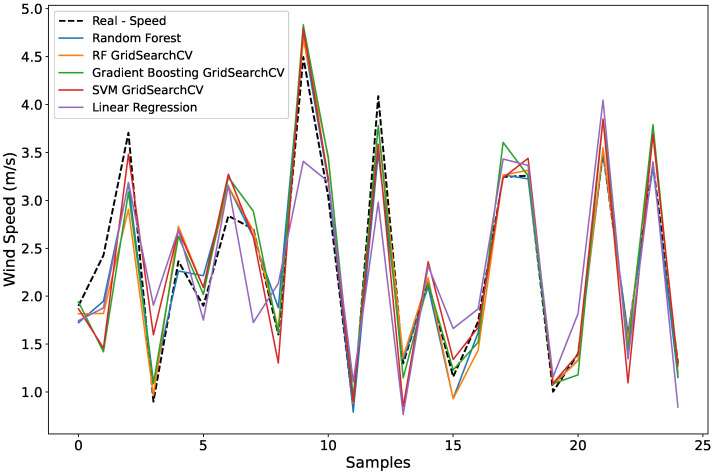
Observed versus predicted wind speed for different machine learning models. The optimized Random Forest (RF) tracks peaks and transitions more closely than others.

Figure 14 shows the comparison between predicted and observed wind speeds. The graph was adjusted to display only 20 samples, making it possible to perform an approximate visual analysis. The optimized RF reproduced the real variations more accurately, including during moments of abrupt changes, while the other models presented predicted values that were higher, lower, or smoother compared to the real data.

A reduction in accuracy during periods of lower thermal variation was common across all approaches, highlighting how the signal to noise ratio of trunk temperature behaves and how it influences prediction sensitivity. As shown in the study by Vassallo et al. [26], atmospheric variables with some physical meaning have an enhanced predictive capacity when combined with thermal measurements.

In general, the optimized RF model stands out, demonstrating excellent ability to handle nonlinear environmental data and different temporal decomposition scales. Its generalization capability in environments with non stationary data indicates its efficiency for wind prediction and environmental monitoring systems.

### 5.3. Wind Direction Forecast

For wind direction, the same methodology applied to wind speed prediction was reproduced, using the same level 0 approximation coefficients from the DWT as the input variable. The performance of each algorithm according to the evaluated metrics is presented in Table 4.

The RF model once again stood out, achieving the lowest error values with MSE = 176.13, MAPE = 6.60%, and the highest coefficient of determination of $R^{2}=0.867$. This result is considered relevant when taking into account the complexity of the problem, since wind direction has an angular and discontinuous behavior, which reduces the stability of predictions. The performance achieved by the RF can be attributed to the ensemble averaging mechanism, ensuring greater resistance to noise and multicollinearity, as discussed by Aminuddin et al. [22]. The authors also highlight that these methods, RF and GB, present notable robustness for handling abrupt directional variations, in situations where model sensitivity becomes crucial.

Similarly, Ekinci et al. [23] observed the flexibility of the algorithm for predictions under seasonal conditions. The good performance of this model is mainly due to its stability during rapid changes in wind direction. Thus, these results reinforce that ensemble-based algorithms are capable of efficiently exploring the most complex information extracted from the DWT.

The GB model again presented performance close to that of RF but with higher sensitivity to abrupt directional transitions. This effect was observed by Oyucu et al. [24], who reported that although GB achieves high accuracy, the model tends to experience temporary instability if not combined with adaptive strategies, such as MLOps pipelines. They suggest that the iterative process of GB can amplify noise at certain transition points instead of capturing the general trend of change.

The SVM, even with kernel adjustment, continued to show difficulties in reproducing the discontinuous wind patterns, especially during rapid variations. This limitation is consistent with the results found by Cao et al. [25], showing that this model performs worse than tree-based approaches when dealing with periodic and nonlinear patterns. It is also important to understand that the fixed kernel function restricts the ability of the SVM to represent rapid angular changes, making it a less suitable model for this task.

Finally, the Linear Regression model obtained the lowest performance, with $R^{2}=0.479$ and MAPE > 14%, confirming its limitations in nonlinear approaches such as atmospheric phenomena [24]. These results reinforce that for the prediction of meteorological variables, it is necessary to apply more flexible models capable of capturing the angular dependence presented by wind.

**Figure 15:**
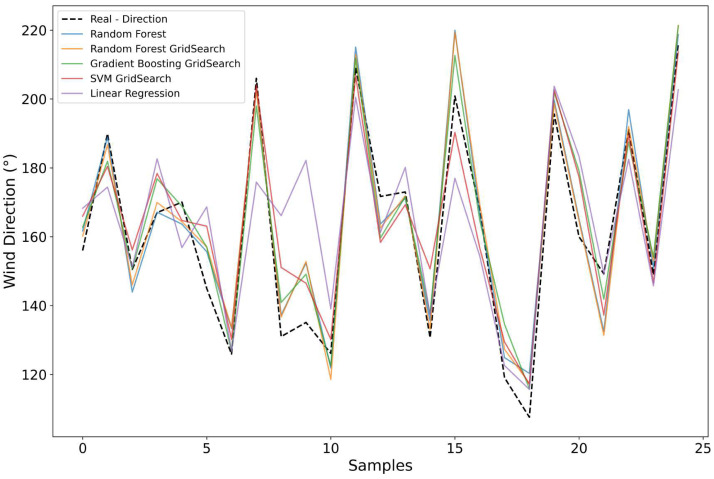
Observed versus predicted wind direction showing the Random Forest (RF) model with the highest fidelity during rapid directional changes.

Figure 15 shows how the predictions behaved in relation to the actual values measured by the meteorological station at the Federal University of Paraíba. The optimized RF model produced more accurate reproduction, especially during moments of sudden change, stabilizing quickly during these transition periods. However, when analyzing points with smaller temperature differences, it is possible to notice a reduction in accuracy.

Similar situations were addressed in the study by Vassallo et al. [26], highlighting the use of physically informed atmospheric variables to increase the ability of models to capture short-term variations and patterns. This shows that it is necessary to integrate additional physical variables or increase the number of sensors to improve the capability of representing small-scale changes.

In general, the results obtained for wind speed and direction show that the combination of the thermal coefficients from the TWD with machine learning methods, especially tree-based models, is an efficient alternative for performing the estimations. Therefore, the methodology applied to the NWS proved to be a promising, low cost, and scalable solution for environmental monitoring and early warning systems in remote regions sensitive to extreme climatic events.

## 6. Conclusions

In the present study, the potential of thermal data collected by sensors installed on a tree to predict wind direction and speed was investigated. The methodology was able to integrate temperature measurements taken at different depths, heights, and cardinal orientations of the trunk with the application of the DWT, so that thermal variability at multiple temporal scales could be captured, from slower and long-term changes to rapid and transient fluctuations. This approach validated the reliability of the internal thermal dynamics of the tree for predicting external meteorological patterns.

The results showed that the use of the DWT allowed the extraction of two sets of coefficients, approximation and detail, with the first set being more effective as inputs for predicting wind variables. The approximation parameters (cA) reflect the stable thermal trends of the dataset, also showing that they are more sensitive to direction, with a stronger Pearson correlation (r≈0.4–0.5) than to wind speed (r≈0.1–0.2). This reinforces the importance of continuously monitoring the thermal gradients around the trunk rather than momentary variations, in order to better represent events related to air movement.

Among all evaluated algorithms, the optimized RF showed the best performance for both wind direction ($R^{2}=0.867$, MAPE = 6.60%) and wind speed ($R^{2}=0.854$, MAPE = 16.44%), demonstrating its robustness when dealing with nonlinear and noisy environmental data. These results are consistent with the studies by Aminuddin et al. [22] and Oyucu and Aksöz [24], which also highlight RF as the most consistent method for wind prediction. The GB model, which achieved the most competitive performance, showed greater sensitivity to data variability, while the SVM and LR models exhibited lower accuracy, especially under highly dynamic conditions. All this evidence reinforces that the RF model is the most suitable for working with the thermal dataset and for the application proposed in this study.

In addition to the efficiency presented by the models, the physical arrangement of the sensors was crucial to correctly capture the trunk’s temperature gradient both vertically and horizontally. The use of the DWT was also proven advantageous for decomposing the time series signal into different scales and capturing the most relevant features for accurate prediction.

The results indicate that the combination of low cost thermal sensing, mathematical modeling, and machine learning algorithms has become a promising approach for real time environmental monitoring. This opens the way for the development of new NWS systems for different regions, including those with limited access or restricted infrastructure.

In the future, we will work to make our model generalizable and subject to more experiments. In a following stage, four instrumented trees will be installed at different sites in João Pessoa (Brazil) to check the replicability of data among species and microclimate conditions. One tree will also be planted at the University of Tacoma, Washington (USA), enabling cross-hemispheric comparisons under different climatic conditions. The expanded application will enable assessment of between-species variation, seasonal effects, and the robustness of the method under different meteorological conditions.

In parallel, future studies will

- Experiment with a wider range of algorithms and hyperparameters, including mixed and deep learning models for better prediction accuracy;
- Fuse complementary data sources, humidity and solar radiation sensors to improve multi-variable correlation analyses;
- Expand across time horizons to better represent seasonal variability and extreme meteorological events.

## Figures and Tables

**Table 1:** Layout of temperature sensors by height, direction, and depth. The label of each sensor follows the pattern T*h***D***e*, where *h* is the height (m), **D** is the direction, and *e* is the depth (cm). For example, T1E9 refers to a sensor installed at 1 m height, on the east side, and 9.0 cm deep into the trunk.

Sensor	Height	Direction	Depth
T1N13	1 m	North	13.5 cm
T1E9	1 m	East	9.0 cm
T1S4	1 m	South	4.5 cm
T2W13	2 m	West	13.5 cm
T2N9	2 m	North	9.0 cm
T2E4	2 m	East	4.5 cm
T3S13	3 m	South	13.5 cm
T3W9	3 m	West	9.0 cm
T3N4	3 m	North	4.5 cm

**Table 2:** Pearson correlation between DWT coefficients (cA, cD) and wind variables (direction and speed).

Sensor	Level	Direction	Speed
T1N13	0	0.5160	0.1800
	1	0.0253	−0.0687
T1E9	0	0.4386	0.1580
	1	−0.0011	0.0141
T1S4	0	0.4191	0.1421
	1	0.0397	0.0320
T2W13	0	0.3686	0.1548
	1	−0.0593	−0.0063
T2N9	0	0.4526	0.1528
	1	0.0187	−0.0234
T2E4	0	0.4514	0.1741
	1	−0.0309	−0.0030
T3S13	0	0.5229	0.1723
	1	0.0068	−0.0128
T3W9	0	0.5147	0.1762
	1	0.0377	0.0085
T3N4	0	0.4271	0.1489
	1	−0.0744	−0.0007

**Table 3:** Wind speed forecasting performance for different models. Random Forest (RF) achieved the best accuracy ($R^{2}$ = 0.854, MAPE = 16.44%).

Model	MSE	$R^{2}$	MAPE
RF (no optimization)	0.189	0.839	18.53%
RF (optimized)	0.172	0.854	16.44%
GB (optimized)	0.232	0.803	20.96%
SVM (optimized)	0.263	0.776	22.33%
LR	0.537	0.544	38.27%

**Table 4:** Wind direction forecasting performance for different models. Random Forest (RF) achieved the lowest MAPE (6.60%) and highest $R^{2}$ (0.867).

Model	MSE	$R^{2}$	MAPE
RF (no optimization)	184.16	0.861	6.83%
RF (optimized)	176.13	0.867	6.60%
GB (optimized)	240.33	0.819	8.16%
SVM (optimized)	460.34	0.653	10.86%
LR	691.35	0.479	14.16%

## Data Availability

The datasets used and analyzed in this study are available from the corresponding author upon reasonable request.

## Abbreviations

The following abbreviations are used in this manuscript:

1-Wire	Single-wire digital communication bus
APC	Article Processing Charge
BMS	Battery Management System
CEAR	Center for Alternative and Renewable Energies
cA	Wavelet approximation coefficients
cD	Wavelet detail coefficients
db4	Daubechies wavelet of order 4
DS18B20	Digital temperature sensor model
DWT	Discrete Wavelet Transform
GB	Gradient Boosting
GIS	Geographic Information System
LoRa	Long Range low-power wide-area radio
LR	Linear Regression
MAPE	Mean Absolute Percentage Error
MODIS	Moderate Resolution Imaging Spectroradiometer
MSE	Mean Squared Error
NWS	Natural Weather Station
QGIS	Quantum Geographic Information System
RBF	Radial Basis Function
RF	Random Forest
$R^{2}$	Coefficient of determination
SVR	Support Vector Regression
SVM	Support Vector Machines
UAV	Unmanned Aerial Vehicle
UFPB	Federal University of Paraíba
WRF	Weather Research and Forecasting model
WRF–SFIRE	WRF coupled with the SFIRE module
